# Dissection of Genetic Effects, Heterosis, and Inbreeding Depression for Phytochemical Traits in Coriander

**DOI:** 10.3390/plants11212959

**Published:** 2022-11-02

**Authors:** Mehrdad Hanifei, Amir Gholizadeh, Mostafa Khodadadi, Shaghayegh Mehravi, Mehnosh Hanifeh, David Edwards, Jacqueline Batley

**Affiliations:** 1 Department of Plant Genetics and Breeding, Faculty of Agriculture, Tarbiat Modares University, Tehran C.P. 14115-336, Iran; 2 Crop and Horticultural Science Research Department, Golestan Agricultural and Natural Resources Research and Education Center, Agricultural Research, Education and Extension Organization (AREEO), Gorgan C.P. 19395-1113, Iran; 3 Seed and Plant Improvement Institute, Agricultural Research Education and Extension Organization (AREEO), Karaj C.P. 33151-31359, Iran; 4 School of Biological Sciences, University of Western Australia, Perth, WA 6009, Australia; 5 Department of Plant Production and Genetics, Faculty of Agriculture, Malayer University, Malayer C.P. 65719-95863, Iran

**Keywords:** coriander, drought stress, general combining ability, gene action, gene pool, F_1_ and F_2_ generations, specific combining ability

## Abstract

Increasing seed yield, fatty acids, and essential oil content are the main objectives in breeding coriander. However, in order to achieve this, there is a need to understand the nature of gene action and quantify the heterosis and inbreeding depression. Towards this, six genetically diverse parents, their 15 F_1_ one-way hybrids, and 15 F_2_ populations were evaluated under different water treatments. The genetic effects of general (GCA) and specific combining ability (SCA) and their interactions with water treatment were significant for five traits. Water deficit stress decreased all traits in both F_1_ and F_2_ generations except for the essential oil content, which was significantly increased due to water deficit stress. Under water deficit stress, a non-additive gene action was predominant in the F_1_ generation, while an additive gene action was predominant in the F_2_ generation for all the traits except seed yield under severe water deficit stress. There was a positive high heterosis for the traits examined in some hybrids. Furthermore, in the F_2_ generation, even after inbreeding depression, some promising populations displayed appropriate mean performance. The results show that the parents used for crossing had a rich, diverse gene pool for the traits studied. Therefore, selection between the individuals of relevant F_2_ populations could be used to develop high yielding hybrids or superior lines.

## 1. Introduction

Coriander (*Coriandrum sativum* L.), a member of the Apiaceae family, is an important medicinal and industrial plant, with applications in the food, drugs, cosmetics, and perfumery industry [1]. Coriander seeds contain both fatty acids and essential oils [2]. The fatty oil composition of coriander seed has previously been characterized [3,4,5,6], showing that petroselinic acid is the main component, with a proportion of 85% of the total fatty acids. In downstream applications, petroselinic acid is broken-down into lauric, adipic, and C_6_ dicarboxylic acids, which are used for synthesizing detergents and nylon polymer [7,8]. Essential oil production in quantities exceeding one ton per year is limited to less than 60 cultivated taxa, including 21 Apiaceae, which includes coriander. Of the 21 commercial Apiaceae producing essential oils, coriander had the highest annual production volume (710 t) and value (USD 49,700,000) in 2016 [9].

The essential oils in coriander are increasingly being recognized as an alternative for other natural components in food [10,11]. They are also used to flavor or remove unpleasant odors of some products in the food industry [12,13]. The essential oil composition of coriander seed has previously been quantified [6,14,15,16], showing that it includes 60–70% linalool, which provides the pleasant characteristics odor [17]). Many medicinal properties have been attributed to coriander essential oil, including antibacterial [18,19], antioxidant [20], antidiabetic [21], anticancer [22], and antimicrobial activities [12,16,23].

It has been previously shown that the amount and composition of substances and secondary metabolites can be affected by water deficit stress in some medicinal plants [24,25]. In some studies, an enhancing effect of water deficit stress on the biosynthesis of essential oils were observed [26,27,28,29,30]. Under stressful growth conditions, secondary metabolite and/or substance production in plants can be enhanced to prevent oxidization in plant cells. Similarly, under water deficit stress, essential oil content may be increased. There is also evidence of water deficit stress decreasing the fatty acid content and yield [31,32,33]. To decrease the adverse effects of drought stress on farmers’ economy, through lower yields of crop plants, cultivation of medicinal plants with improved potential of secondary metabolites production under drought-affected areas could be suggested as an alternative [26].

Production of drought tolerant coriander genotypes with high seed yield and essential and fatty oil contents are possible through plant breeding. However, a successful plant improvement program relies on an understanding of the nature of the gene action involved in the inheritance of that traits under target growth condition. Griffing’s [34] diallel analysis has been used to uncover the behavior of gene actions involved in the controlling of traits. This method has also been used to estimate general combining ability (GCA) and specific combining ability (SCA) variances in both self-pollinated and open-pollinated crop groups [9,35,36,37,38,39,40,41,42,43,44,45]. Furthermore, diallel analysis on F_1_ crosses has previously been done to estimate genetic parameters and combining ability in coriander [9].

Heterosis in F_1_ hybrids can reflect the SCA and GCA of relevant parents. On the other hand, inbreeding depression measures the amount of vigor reduction in segregating generations due to self-pollination [46]. Investigation of heterosis in coriander is important for the plant breeder to identify the superior crosses in the first generation itself. Furthermore, the magnitude of heterosis provides a basis for determining genetic diversity and also serves as a guide to the choice of desirable parents. However, we are not aware of any published research on heterosis and inbreeding depression of coriander seed yield or its seed quality related traits. Therefore, the present study was carried out to recognize suitable cross combinations from adapted parents for commercial exploitation of seed yield, essential and fatty oil content, and their utilization in future improvement programs under different watering conditions.

## 2. Results and Discussion

### 2.1. Combined Analysis of Variance for Traits under Water Treatments

The combined analysis of variance revealed the presence of a significant difference between water treatments for all of traits in both F_1_ hybrids and F_2_ generations (Table 1). These observations indicate that the parental selection for diallel crosses was appropriate. Along with the main water treatment and genotype effects, the genotype × water treatment interaction effect was significant for all traits in both F_1_ hybrids and F_2_ generations (Table 1). This significant genotype (F_1_ hybrids + F_2_ generations) × water treatment interaction reflects a different growth response of genotypes in differently watered growth conditions.

Analysis of variance for genetic effects revealed that both additive and non-additive gene actions are involved in the expression of traits in both F_1_ hybrids and F_2_ generations. Furthermore, the significant GCA × water treatment and SCA × water treatment interactions effect for all traits in both F_1_ and F_2_ generations (Table 1) reveals that the general combining ability of parents and specific combining ability of hybrids were differently determined by additive and non-additive gene actions under different water treatments, respectively. Therefore, selection for parents with high GCA or a hybrid with high SCA should be done according to the conditions of the target cultivating environment.

### 2.2. Effect of Water Deficit Stress on Measured Traits

Generally, the results indicated that seed yield, essential oil yield, fatty oil content, and fatty oil yield were negatively affected by water deficit stress in both F_1_ hybrids and F_2_ generations in coriander. However essential oil content was significantly increased under water deficit stress (Table 2).

#### 2.2.1. Effect of Water Deficit Stress on Seed Yield

As shown in Table 2, seed yield was significantly affected by water treatments. The highest seed yield was obtained in well-watered conditions while the minimum seed yield was obtained in severe water deficit stress in both F_1_ hybrids and F_2_ generations. A reduction in seed yield of coriander under water deficit conditions was also reported by Nadjafi et al. [47] and Khodadadi et al. [9].

In other aromatic and medicinal crops, similar results were observed by Zehtab-Salmasi et al. [48] in dill (*Anethum graveolens* L.), Bannayan et al. [49] in *Plantago ovata* and *Nigella sativa*, Laribi et al. [50] in caraway (*Carum carvi* L.), Ekren et al. [51] in purple basil (*Ocimum basilicum* L.), and Alinian and Razmjoo [52] in cumin (*Cuminum cyminum* L.) under drought stress conditions. A seed yield reduction under drought stress is thought to occur through insufficient photosynthesis due to stomata closure and thereafter a reduction in CO_2_ uptake [53], shortening flowering and seed setting periods, and preferential allocation of assimilates to the roots rather than the shoots [52].

#### 2.2.2. Effect of Water Deficit Stress on Essential Oil Content and Essential Oil Yield

The largest value of essential oil content was obtained in the moderate water deficit stress while the lowest essential oil content was recorded in well-watered for both F_1_ hybrids and F_2_ generations. This suggests that drought stress has a positive effect on the essential oil content in coriander. An increase in the essential oil content under drought stress has also been documented by Baher et al. [54] in *Satureja hortensis* L, Yassen et al. [55] in *Ocimum basilicum* L., Omidbaigi et al. [56] in *Ocimum basilicum*, Nurhan and Vazquez [57] in *Lippia graveolens* and *Lippia berlandieri* Schauer, Khalid [58] in *Ocimum basilicum* L. and *Ocimum americanum* L., Petropoulos et al. [25] in *Petroselinum crispum* [Mill.], Bettaieb et al. [31] in *Salvia officinalis* L., Ekren et al. [51] in *Ocimum basilicum* L., and Alinian et al. [26] in *Cuminum cyminum* L.

However, drought stress led to a decrease in essential oil yield in both F_1_ hybrids and F_2_ generations (Table 2). The highest value of essential oil yield was obtained in the well-watered condition and the lowest essential oil yield was observed in severe water deficit stress for both F_1_ hybrids and F_2_ generations (Table 2). Similar results were reported by Alinian and Razmjoo [52], Hossein et al. [59], Munnu and Ramesh [60], and Zehtab-Salmasi et al. [48]. Essential oil yield depends on essential oil content and seed yield. As the drought stress caused a higher reduction in seed yield than an increase in essential oil content, essential oil yield was reduced under water deficit stress conditions [59].

#### 2.2.3. Effect of Water Deficit stress on Fatty Oil Content and Yield

The largest fatty oil content and yield values were obtained in well-watered conditions and the least fatty oil content and fatty oil yield values were obtained under severe water deficit stress for both F_1_ hybrids and F_2_ populations. Similarly, Munnu and Ramesh [60] in rosemary, Zehtab-Salmasi et al. [48] in dill (*Anethum graveolens* L.), Hamrouni et al. [33] in safflower (*Carthamus tinctorius* L.), Bettaieb et al. [31] in sage (*Salvia officinalis* L.), and Bettaieb et al. [32] in cumin (*Cuminum cyminum* L.) observed a significant decreasing effect of water deficit stress on fatty oil content and fatty oil yield.

### 2.3. Nature of Gene Action

The significant GCA and SCA variances for all traits in both F_1_ hybrids and F_2_ populations indicate that both additive and non-additive gene actions contributed to determine these traits. Khodadadi et al. [9] also reported that both non-additive and additive gene actions for the inheritance of different traits are important in coriander. The GCA/SCA ratio reflects the degree of the trait that is transmitted from the parents to the progeny. When the GCA/SCA ratio is closer to one and zero it shows that the additive and non-additive gene actions are mostly involved in inheritance of the trait, respectively. Considering the GCA/SCA ratio observed in this study, non-additive gene action was predominant for seed yield, essential oil yield, and fatty oil yield traits in F_1_ and F_2_ generations under well-watered conditions (Table 3). This kind of situation can arise due to the repulsion phase linkage between different genes involved in the inheritance of these traits [61].

In advanced generations, when a coupling linkage is present, additive genetic variance decreases and when the repulsion linkage is present, additive genetic variance increases [62]. Therefore, to improve seed yield, essential oil yield, and fatty oil yield traits under well-watered conditions, selection should be delayed to the later generations of segregation.

For fatty oil content, non-additive gene action was predominant in F_1_ hybrids, while in F_2_ generations the additive genetic effects were more important under well-watered conditions (Table 3). The difference in F_1_ and F_2_ results is due to the breakdown of linkages between genes and an increase in frequency of homozygous loci and decrease of heterozygous loci. Furthermore, essential oil content was predominantly governed by additive gene action in both the F_1_ hybrids and F_2_ generations. The presence of mostly additive gene action in F_2_ generations for fatty oil content and in both F_1_ and F_2_ generations for essential oil content suggests that selection programs can be effective in the F_2_ and later generations for improvement of fatty oil content and essential oil content traits under well-watered conditions.

In severe water deficit stress, the results of the GCA/SCA ratio for seed yield showed that a non-additive gene action was predominant in both the F_1_ hybrids and F_2_ populations (Table 3). Therefore, to improve seed yield under severe water deficit stress conditions, selection should be delayed to the later generations of segregation for loss of non-additive gene actions. For seed yield under moderate water deficit stress and essential oil content, fatty oil content, essential oil yield and fatty oil yield under both moderate and severe water deficit stress conditions, the non-additive gene action in F_1_ hybrids while an additive gene action in F_2_ generation were more important (Table 3). Therefore, breeding programs based on selection can be effective in the F_2_ and later generations for improvement of these traits under water deficit stress.

### 2.4. Mean Performance, Heterosis, and Inbreeding Depression

#### 2.4.1. Seed Yield

**Figure 1:**
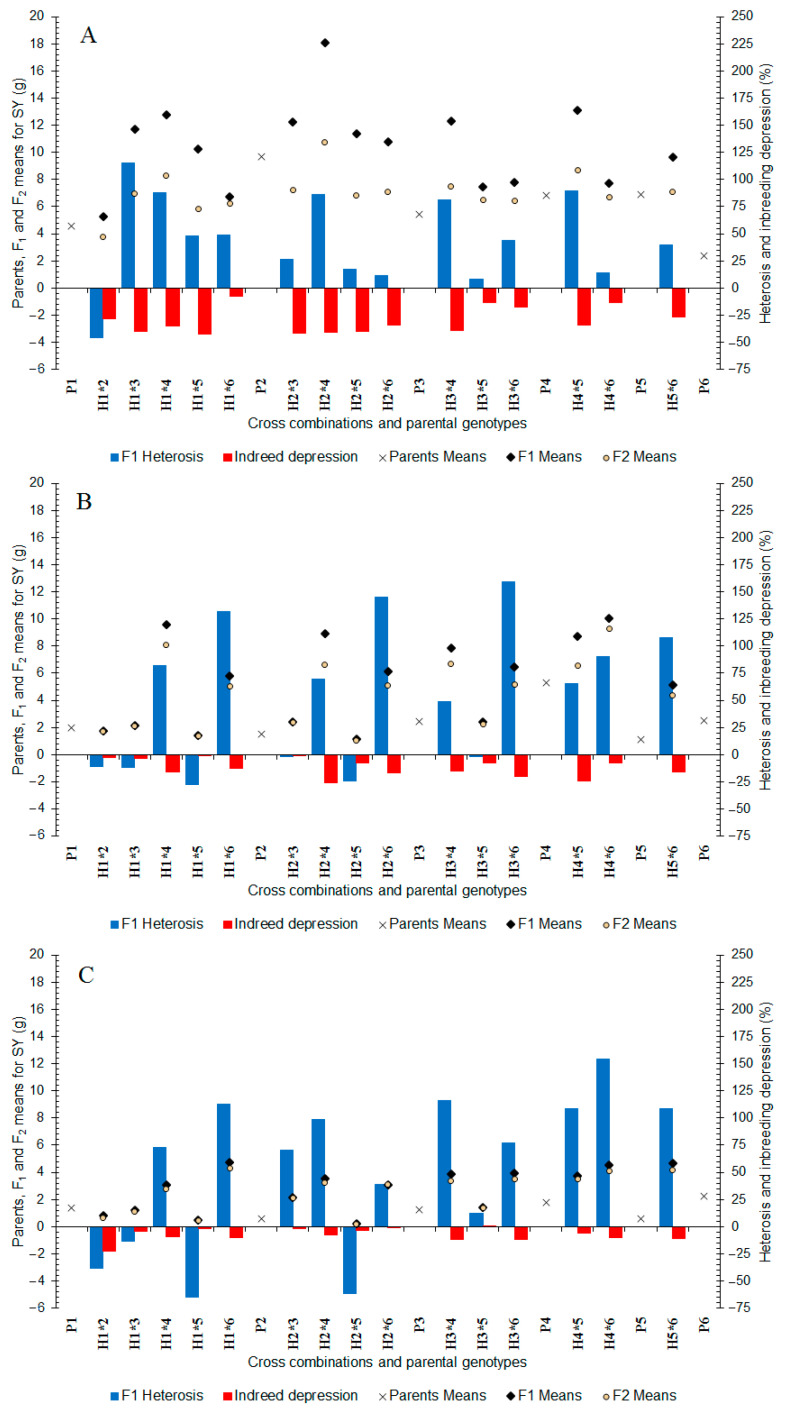
Mean, heterosis, and inbreeding depression for seed yield in F_1_ and F_2_ generations of coriander crosses. (**A**) Well-Watered, (**B**) Moderate Water Stress, and (**C**) Severe Water Stress. P_1_–P_6_: six parental coriander genotypes; H_1×2_–H_5×6_: 15 half-diallel hybrids.

In well-watered conditions, seed yield varied from 2.40 (P_6_) to 9.71 g (P_2_) between the parents and ranged from 5.26 to 18.10 g (H_2×4_) between the F_1_ hybrids (Figure 1A). Parental genotypes of the H_2×4_ had approximately half the yield (6.80–9.71 g) compared to their hybrids. In the F_2_ generation, the seed yield varied from 3.75 to 10.71 g between the hybrids (Figure 1A). Similar to the F_1_ generation, in the F_2_ the highest seed yield was obtained by H_2×4_. In addition, in the F_1_ generations, almost all hybrids exhibited positive heterosis (7.82–115.40%) in which P_4_ involved hybrids mostly showed high heterosis (+80.91 to +89.74%). Inbreeding depression from F_1_ hybrids to F_2_ generations ranged from −7.94 to −42.80% for seed yield (Figure 1A).

In moderate water deficit stress conditions, the seed yield varied from 1.14 (P_5_) to 5.27 g (P_4_) between the parents and ranged from 1.17 to 10.03 g between the F_1_ hybrids (Figure 1B). A high seed yield was obtained in five F_1_ hybrids including H_4×6_ (10.03 g), H_1×4_ (9.58 g), H_2×4_ (8.93 g), H_4×5_ (8.71 g) and H_3×4_ (8.85 g). In the F_2_ generation, the seed yield varied from 1.08 to 9.29 g (Figure 1B). F_2_ generations relevant to the high yielding F_1_ hybrids also exhibited the highest seed yield. When P_4_ and P_6_ contributed as one of the mating partners, the highest heterosis vigor was obtained (+107.40 to +159.59%). Inbreeding depression from F_1_ hybrids to F_2_ populations had a larger range for seed yield (−0.36 to −26.05%) in moderate water deficit stress than well-watered conditions (Figure 1B).

In severe water deficit stress, the seed yield varied from 0.58 (P_5_) to 2.24 g (P_6_) between the parents and from 0.22 to 4.77 g between the F_1_ hybrids (Figure 1C). In the F_2_ generation, seed yield varied from 0.21 to 4.28 g (Figure 1C) and a high seed yield obtained from F_2_ populations was derived from the hybrids that P_4_ and P_6_ contributed to. The heterosis values for seed yield ranged between −64.68 and +154.54% (Figure 1C) and many of the hybrids exhibited positive heterosis. Similar to moderate water deficit stress, inbreeding depression from F_1_ hybrids to F_2_ populations in severe water stress showed a larger range (−0.59 to −22.66%) than well-watered conditions (Figure 1C).

Higher heterosis and lower inbreeding depression in water deficit stressed conditions than those in well-watered conditions reveal that the respective parents of the hybrids were most likely carriers of drought tolerance alleles, which could be homozygous recessive [63]. Therefore, their hybrids appeared superior in water deficit stressed conditions compared with the high yielding hybrids, which were superior in well-watered conditions. In case of inbreeding depression from F_1_ hybrids to F_2_ generations, heterozygosity will be at the maximum 50% of alleles. Therefore, an appearance of drought tolerance in F_2_ generations could be kept by using heterozygous genes.

#### 2.4.2. Essential Oil Content

**Figure 2:**
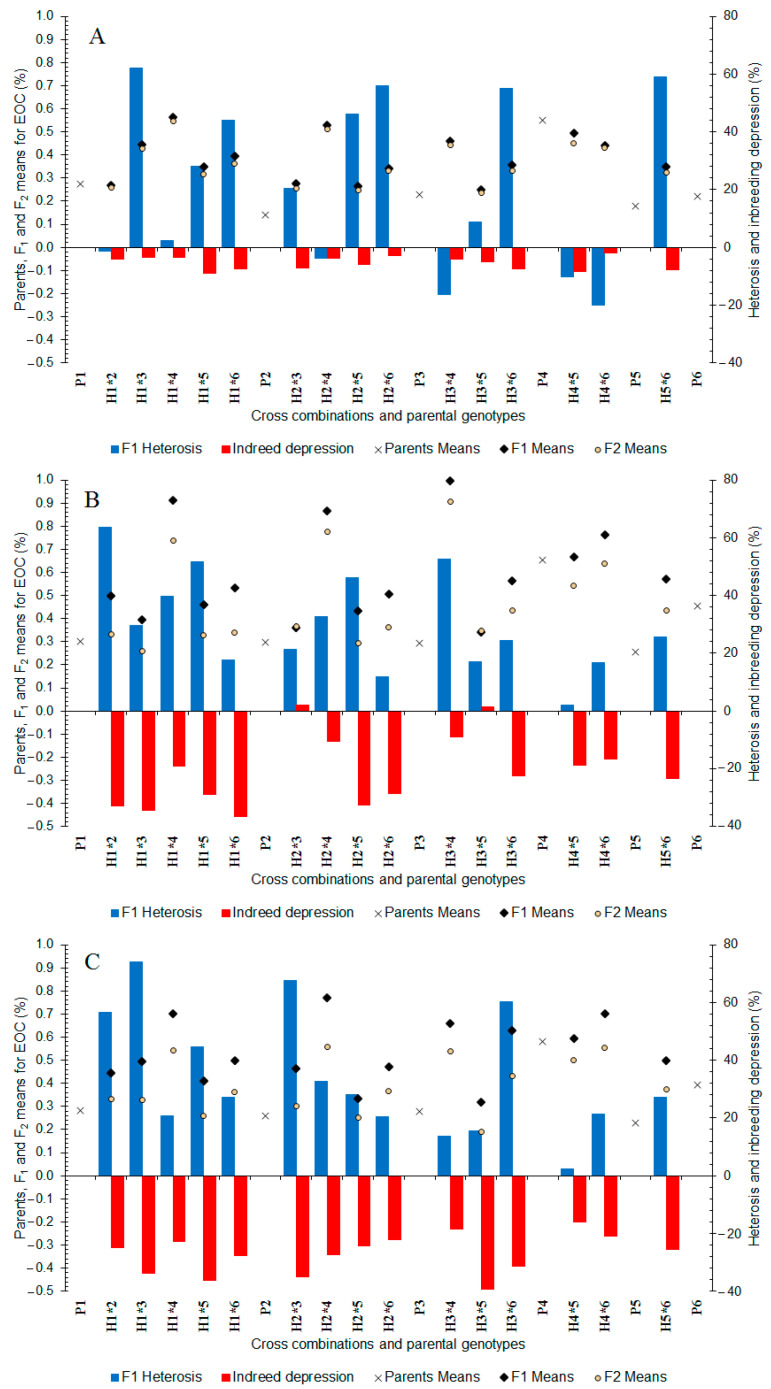
Mean, heterosis, and inbreeding depression for essential oil content in F_1_ and F_2_ generations of coriander crosses. (**A**) Well-Watered, (**B**) Moderate Water Stress, and (**C**) Severe Water Stress. P_1_–P_6_: six parental coriander genotypes; H_1×2_–H_5×6_: 15 half-diallel hybrids.

In well-watered treatments, the essential oil content ranged from 0.140% (P_2_) to 0.550% (P_4_) between the parents and from 0.250 to 0.563% between the F_1_ hybrids (Figure 2A). The highest essential oil content was obtained in five hybrids of P_4_ (0.440–0.563%), followed by the H_1×3_ hybrid. In the F_2_ generation, essential oil content ranged from 0.237 to 0.545% (Figure 2A) and five of the F_2_ populations which had P_4_ as one of the mating partners exposed the highest essential oil content (0.431–0.545%). In the F_1_ generation (Figure 2A), many of the hybrids showed positive heterosis (+2.42 to +62.20%). In addition, all the F_2_ populations showed inbreeding depression (−2.07 to −9.06%) (Figure 2A).

In moderate water deficit stress, the essential oil content ranged from 0.257% (P_5_) to 0.653% (P_4_) between the parents and from 0.343 to 0.997% between the F_1_ hybrids (Figure 2B). The highest essential oil content was recorded in five hybrids derived from P_4_ (0.667–0.997%). In the F_2_ generation, essential oil content ranged from 0.258 to 0.907% between the populations (Figure 2B) and, similar to the F_1_ hybrids, five populations derived from P_4_ showed the highest essential oil content (0.542–0.907%). In the F_1_ generation, all crosses exposed positive heterosis (+2.04 to +63.74%) (Figure 2B). Furthermore, almost all the F_2_ populations showed inbreeding depression (−9.00 to −36.52%) (Figure 2B).

In severe water deficit stress, the essential oil content ranged from 0.227% (P_5_) to 0.580% (P_4_) between the parents and from 0.320 to 0.770% between the F_1_ hybrids (Figure 2C). The highest essential oil content was obtained in the five hybrids of P_4_ (0.593–0.770%). In the F_2_ generation, the essential oil content was 0.191–0.560% between the cross populations (Figure 2C) and five derivatives of P_4_ showed high essential oil content (0.499–0.560%). In the F_1_ generation, all hybrids showed positive heterosis (+2.30 to +74.12%) and all of the F_2_ populations showed inbreeding depression (−15.89 to −40.38%) (Figure 2C).

The ranges of heterosis and inbreeding depression were higher in water deficit stressed conditions compared to the well-watered conditions. Generally, high heterosis along with high inbreeding depression refers to the presence of genes with non-additive action and high heterosis and the lowest inbreeding depression indicates the presence of genes with additive action [64]. Low inbreeding depression in well-watered conditions suggests that increased vigor of F_1_s in such cases are expected to be mainly due to an accumulation of favorable additive action genes. In addition, high inbreeding depression in water deficit stress conditions indicates that non-additive action genes play a major role in the inheritance of essential oil content. Our results are in accordance with previous research on inbreeding depression under water deficit stressed conditions [65,66]. In F_2_, even after inbreeding depression, some crosses exhibited good performance, indicating the potential of these crosses for the development of high essential oil content cultivars. The derivatives of the P_4_ parent displayed better mean performance as compared to other parents, even after segregation and inbreeding depression. Therefore, the P_4_ population could be used in the segregating generations to obtain genotypes with high essential oil content under different water treatments.

#### 2.4.3. Fatty Oil Content

**Figure 3:**
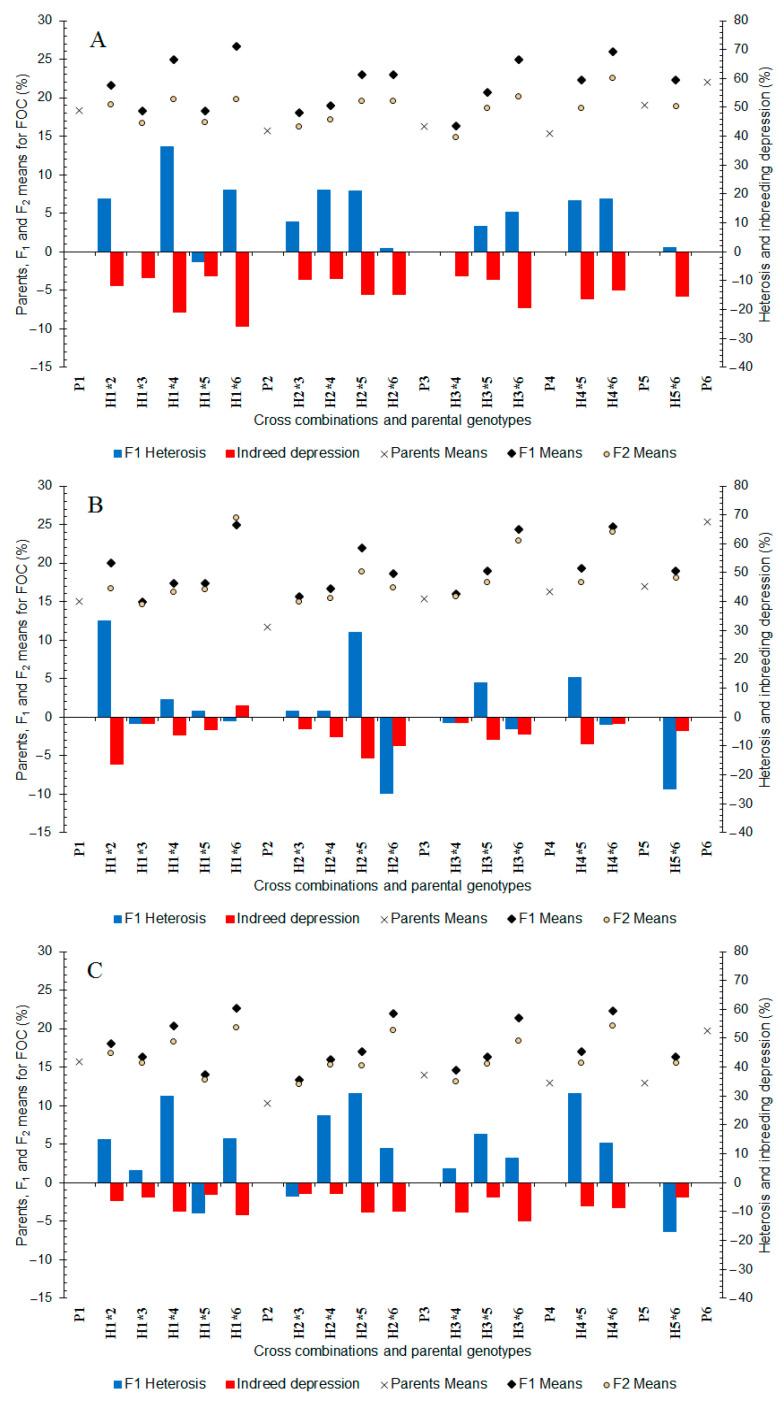
Mean, heterosis, and inbreeding depression for fatty oil content in F_1_ and F_2_ generations of coriander crosses. (**A**) Well-Watered, (**B**) Moderate Water Stress, and (**C**) Severe Water Stress. P_1_–P_6_: six parental coriander genotypes; H_1×2_–H_5×6_: 15 half-diallel hybrids.

In well-watered conditions, the fatty oil content varied from 15.33 (P_4_) to 22% (P_6_) between the parents and ranged from 16.33 to 26.67% between the F_1_ hybrids (Figure 3A). The highest fatty oil content was recorded for hybrids of P_6_ (H_1×6_ (26.67%), H_4×6_ (26.0%), H_3×6_ (25.0%), and H_2×6_ (23.0%)) followed by H_1×4_ hybrid. The parental genotypes of these promising hybrids also had high fatty oil content (18.33–22.0%). In the F_2_ generation, the fatty oil content varied from 14.94 to 22.54% between the populations (Figure 3A). The highest fatty oil content was obtained in the F_2_ generation by P_6_ hybrids and followed H_1×4_, H_2×5_, H_1×2_ hybrids. In the F_1_ generation, heterosis ranged from +0.00 to +36.36% for fatty oil content (Figure 3A) and in the F_2_ generation, inbreeding depression for fatty oil content was observed from −8.32 to −25.75% (Figure 3A).

In moderate water deficit stress, the fatty oil content varied from 11.67 (P_2_) to 25.33% (P_6_) and 15.00 to 25.0% between parents and F_1_ hybrids, respectively (Figure 3B). The highest fatty oil content was observed in eight F_1_ hybrids derived from P_6_. In the F_2_ generation, the fatty oil content varied from 14.68 to 25.98% between hybrids (Figure 3B) and the highest fatty oil content (22.89–25.98%) was recorded for three hybrids of P_6_. The heterosis values for fatty oil content were +1.96 to +33.33% (Figure 3B) and almost all hybrids showed positive heterosis. F_2_ populations showed inbreeding depression for fatty oil content (−2.03 to −16.37%) (Figure 3B).

In severe water deficit stress, the fatty oil content varied from 10.33 (P_2_) to 19.67% (P_6_) and 13.33 to 22.67% between parents and F_1_ hybrids, respectively (Figure 3C). The highest fatty oil content was recorded in F_1_ hybrids involving P_6_, followed by the H_1×4_ hybrid. In the F_2_ generation, fatty oil content varied from 12.85 to 20.41% between the hybrids (Figure 3C) and the highest fatty oil content was obtained from hybrids of P_6_. The heterosis values for fatty oil content ranged from +4.26 to +30.77% (Figure 3C) and many hybrids showed positive heterosis. The F_2_ generations displayed inbreeding depression (−3.64 to −13.30%) for fatty oil content (Figure 3C). Overall, it was revealed that F_2_ populations from P_6_ could be utilized for developing cultivars with high fatty oil content under different water treatments.

The ranges of heterosis and inbreeding depression were higher in well-watered than water stressed conditions. High heterosis is well-known to be a result of the effects of non-additive genes [67,68,69]. Therefore, the higher heterosis and inbreeding depression in well-watered condition suggest that non-additive gene actions were more predominant in well-watered conditions compared to the water deficit stress conditions. F_2_ progenies derived from P_6_ contributed hybrids showed better mean performance even after inbreeding depression than their parents, indicating the presence of transgressive segregation for fatty oil content under different water treatments.

#### 2.4.4. Essential Oil Yield and Fatty Oil Yield

**Figure 4:**
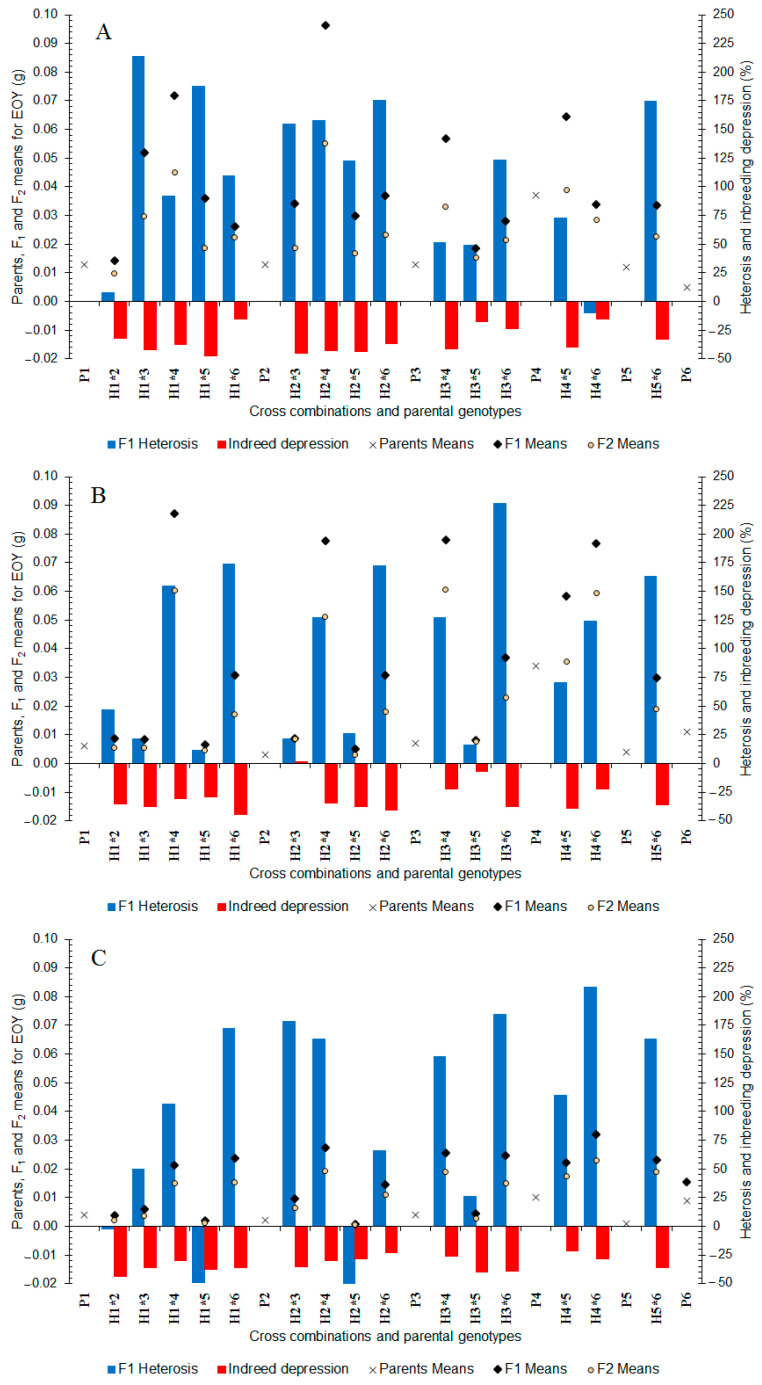
Mean, heterosis, and inbreeding depression for essential oil yield in F_1_ and F_2_ generations of coriander crosses. (**A**) Well-Watered, (**B**) Moderate Water Stress, and (**C**) Severe Water Stress. P_1_–P_6_: six parental coriander genotypes; H_1×2_–H_5×6_: 15 half-diallel hybrids.

In well-watered treatments, the essential oil yield ranged from 0.005 (P_6_) to 0.037 g (P_4_) among the parents and from 0.014 to 0.096 g between the F_1_ hybrids (Figure 4A). High essential oil yield was obtained for four P_4_ crosses (0.057–0.096 g). In the F_2_ generation, essential oil yield ranged from 0.010 to 0.055 g between the cross generations (Figure 4A) and four crosses of P_4_ showed a high essential oil yield (0.033–0.055 g). In the F_1_ generation (Figure 4A), almost all crosses indicated positive heterosis for essential oil yield (+7.48 to +213.91%). In addition, all of the F_2_ populations showed inbreeding depression (−15.06 to −47.80%) (Figure 4A).

In moderate water stress, the essential oil yield ranged from 0.003 (P_2_) to 0.034 g (P_4_) between the parents and from 0.005 to 0.087 g between the F_1_ hybrids (Figure 4B). The highest essential oil yield was recorded for five P_4_ crosses (0.058–0.087 g), followed by H_1×6_, H_3×6_, H_5×6_ hybrids. In the F_2_ generation, essential oil yield ranged from 0.003 to 0.061 g between the cross population (Figure 4B) and, similar to the F_1_ generation, crosses of P_4_ showed the highest essential oil yield (0.036–0.061 g). In the F_1_ generation, all crosses showed positive heterosis (+11.22 to +226.33%) (Figure 4B). In addition, almost all of the F_2_ populations showed inbreeding depression for essential oil yield (−6.88 to −44.40%) (Figure 4B).

In severe water stress, the essential oil yield ranged from 0.002 (P_5_) to 0.010 g (P_4_) between the parents and from 0.001 to 0.032 g between the F_1_ hybrids (Figure 4C). The highest essential oil yield was obtained in crosses of P_4_ (0.021–0.032 g), followed by H_1×6_, H_3×6_, H_5×6_ hybrids. In the F_2_ generation, essential oil yield ranged from 0.001 to 0.023 g between the cross generations (Figure 4C) and progenies of P_4_ and P_6_ showed the highest essential oil yield. In the F_1_ generation, almost all crosses displayed positive heterosis (+26.01 to +208.31%) (Figure 4C). The F_2_ generation showed inbreeding depression (−21.96 to −40.85%) (Figure 4C). Overall, the results indicated that P_4_ population could be used in the segregating generations to obtain genotypes with essential oil yield potential under different water treatments.

**Figure 5:**
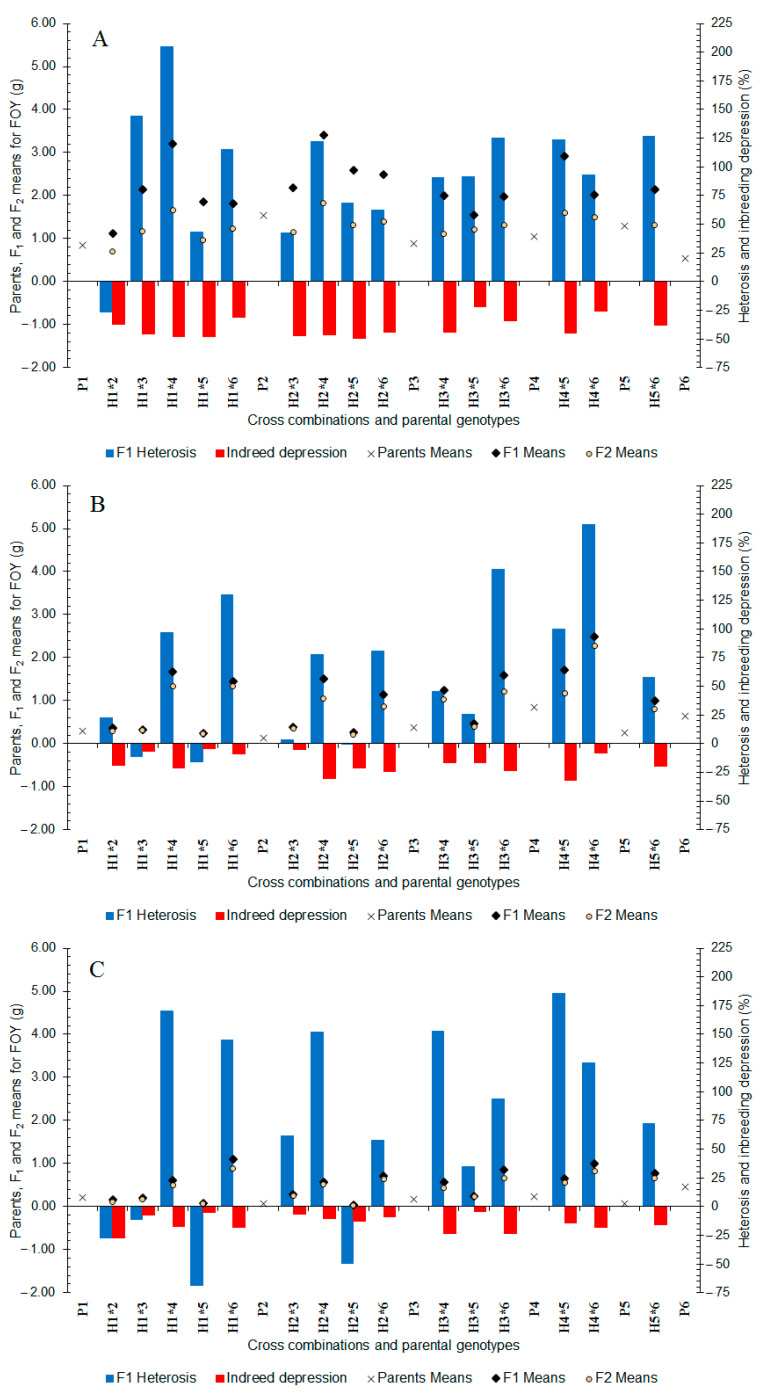
Mean, heterosis, and inbreeding depression for fatty oil yield in F_1_ and F_2_ generations of coriander crosses. (**A**) Well-Watered, (**B**) Moderate Water Stress, and (**C**) Severe Water Stress. P_1_–P_6_: six parental coriander genotypes; H_1×2_–H_5×6_: 15 half-diallel hybrids.

In well-watered conditions, the fatty oil yield varied from 1.12 to 3.41 g between parents and F_1_ hybrids (Figure 5A). The highest fatty oil yield was obtained from H_2×4_, H_1×4_ hybrids. In the F_2_ generation, the fatty oil yield varied from 0.71 to 1.82 g between the generations (Figure 5A) and the highest fatty oil yield was recorded in generations derived from the hybrids of P_4_. The heterosis values for fatty oil yield ranged from −26.95 to +204.96% (Figure 5A) and all hybrids showed positive heterosis. F_2_ populations displayed inbreeding depression for fatty oil yield (−21.88 to −49.31%) (Figure 5A).

In moderate water stress, the fatty oil yield ranged from 0.13 (P_2_) to 0.85 g (P_4_) between the parents and from 0.24 to 2.48 g between the F_1_ hybrids (Figure 5B). High values of fatty oil yield were recorded in hybrids involving P_4_ and P_6_. In the F_2_ generation, fatty oil yield ranged from 0.20 to 0.2.27 g between the cross generations (Figure 5B) and the crosses of P_4_ and P_6_ showed high fatty oil yield. In the F_1_ generation (Figure 5B) almost all of the hybrids showed positive heterosis (+3.42 to +191.18%). In addition, almost all of the F_2_ population showed inbreeding depression (−4.14 to −31.64%) (Figure 5B).

In severe water stress, the fatty oil yield varied from 0.06 (P_2_) to 0.45 g (P_6_) and 0.04 to 1.04 g between parents and F_1_ hybrids, respectively (Figure 5C). High values of the fatty oil yield were recorded in F_1_ hybrids involving P_6_, followed by hybrids of P_4_. In the F_2_ generation, fatty oil yield varied from 0.03 to 0.89 g between the generations (Figure 5C) and high values of the fatty oil yield was obtained from hybrids of P_6_. The heterosis values of fatty oil yield ranged from +35.04 to +185.27% (Figure 5C) and many of the hybrids showed positive heterosis. The F_2_ populations showed inbreeding depression (−4.53 to −27.02%) (Figure 5C). Overall, the results indicated that P_6_ and P_4_ populations could be used in the segregating generations to obtain genotypes with high fatty oil yield potential under different water treatments.

Inbreeding depression was higher in well-watered conditions compared to water deficit stressed conditions for essential oil yield and fatty oil yield, indicating that inbreeding depression was unstable across environments. In addition, the results revealed the higher heterosis values for essential oil yield and fatty oil yield than other traits, indicating that non-additive genes were more responsible for the expression of these traits. These findings can be confirmed by the results of the GCA/SCA ratio in Table 3.

The utilization of hybrid vigor is one of the ways to improve yield in plant breeding. The existence of a considerable degree of natural outcrossing has made it possible to use genetic diversity through the production of heterotic hybrids [70]. In coriander, heterosis cannot be exploited for higher production through commercial hybrids due to the nature of flowering and poor seed recovery during hybridization.

However, estimation of heterosis for seed yield, fatty oil, and essential oils content will help in identifying crosses that can lead to the development of advanced, promising lines in segregating generations in coriander. Furthermore, estimation of heterosis coupled with inbreeding depression shows whether an amount of the vigor observed in segregating generations can be fixed in later generations by self-pollinating [46,71]. The results showed that there was a positive heterosis for the traits examined in coriander, which is evidence for the existence of potential heterosis in Iranian

In the present study, the significant SCA effect indicates that there was a non-additive gene effect, which could be the cause of the heterosis on the progenies observed and the selection will not be effective in early generations. Hence, selection could be practiced in advance generations confirming to earlier reports.

The results showed that many of the F_2_ populations had inbreeding depression, and this was higher for seed yield, essential oil yield, and fatty oil yield. Inbreeding depression of hybrids with high yield were higher than hybrids with low and moderate. Khan et al. [40,72] and Soomro and Kalhoro [73] reported that F_1_ hybrids with high performance were also correlated with higher inbreeding depression. Showing heterosis in F_1_ and inbreeding depression in F_2_ reveals the nature of gene action involved in the expression of the vigor in F_1_ and depression in F_2_. In F_2_ generations, the offspring of the parental genotypes P_4_ and P_6_ displayed better mean performance as compared to their parents and the selection in these crosses can provide transgressive gene recombinants for the studied traits. P_4_ and P_6_ crosses are required to be subjected to the pedigree/progeny selection directly for reaching the high potential cultivars. In addition, P_4_ and P_6_ parents can be used as source of elite parents for synthetic cultivars [40,72] in coriander.

## 3. Materials and Methods

### 3.1. Plant Material and Growth Conditions

Genotypes used for making diallel crosses were evaluated in a preliminary experiment for drought tolerance by Khodadadi et al. [74]. The characteristics of selected parental genotypes are summarized in Table 4. All the six parents contributed to the production of 15 F_1_ hybrids (without reciprocals) through a half diallel mating system in 2015. Some of these F_1_ hybrids’ seeds were used to produce 15 F_2_ generations through self-pollination. All of the six parents, 15 F_1_ hybrids, and 15 F_2_ generations were evaluated under three levels of irrigation regimes. A field trial consisted of three experiments close together at a distance of 1 m. These experiments were well-watered (WW), moderate water deficit stress (MWDS), and severe water deficit stress (SWDS). Each of these experiments were carried out through a randomized complete block design, with three replications, at the research field of Tarbiat Modares University (51°09′ E; 35°44′ N; altitude 1265 m), Tehran, Iran during the growing season of 2017. In the WW experiment, a set of genotypes were well-watered over the entire experiment period. In the MWDS experiment, a set of genotypes were well-watered until the end of the flowering stage, at which point one recovery watering was applied. In the SWDS experiment, watering was similar to the WW experiment until an appearance of the flowering stage, after which watering was cut off completely. The research field soil physical and chemical characteristics are presented in Table 5.

### 3.2. Traits Measurements

The phytochemical traits measured include essential oil content (EOC), fatty acid content (FAC), essential oil yield (EOY), fatty acid yield (FAY), and seed yield per plant (SY). For measuring the seed yield of parents and relevant F_1_ hybrids, 10 plants were harvested from each of the experimental plots. In the F_2_ generations, 30 plants were harvested from each of the experimental plots. For extracting the essential oil, 30 g of dried coriander seeds were well powdered and subjected to hydro-distillation in Clevenger-type apparatus for 120 min. Essential oil content (%*w*/*w*) was computed through the weight (g) of essential oil per 100 g of seed [9].

Essential oil yield was computed through multiplying the essential oil content by seed yield per plant (g). For measuring fatty acid content, 2 g of powdered seed sample of coriander were subjected to soxhlet-apparatus with 250 mL of petroleum ether for 6 h. Fatty acids were removed after mixture filtration and solvent evaporation under reduced temperature and pressure [9,52]. Finally, fatty acid yield was estimated by multiplying fatty acid content with seed yield per plant (g) for each plot.

### 3.3. Statistical Analysis

The datasets were firstly tested for normality using the Anderson and Darling normality test [75]. The analysis of variance for GCA and SCA effects were done according to Griffing‘s [34] method 2, model 1 using a SAS program suggested by Ref. [76]. The mean values of traits in water treatments were compared using the least significant difference (LSD) method at 5% probability level. Estimates of $\sigma_{g}^{2}$ (general combining ability variance) and $\sigma_{s}^{2}$ (specific combining ability variance) were computed according to the random-effects model [76]. The GCA/SCA ratio was computed according to the method proposed by Baker [77] (Equation (1)).

(1) $$\mathrm{GCA}/\mathrm{SC}A_{\mathrm{ratio}}=\frac{2\sigma_{g}^{2}}{2\sigma_{g}^{2}+\sigma_{s}^{2}}$$

The best parent heterosis was calculated in F_1_ hybrids using the formula suggested by Fonseca and Patterson [78] (Equation (2)).

(2) $$\mathrm{Heterosis}=\frac{F_{1}-\mathrm{BP}}{\mathrm{BP}}$$

where F_1_ and BP are target hybrid and best parent values, respectively. In addition, the observed inbreeding depression (ID) was estimated as a percent of the decrease in F_2_ mean when compared with F_1_ hybrid mean according to the formula suggested by Khan et al. [40] (Equation (3)). The ${\bar{F}}_{1}$ is the mean value of F_1_ hybrid and ${\bar{F}}_{2}$ is the mean value of F_2_ generations mean of parents.

(3) $$\mathrm{ID}(\%)=\frac{{\bar{F}}_{2}-{\bar{F}}_{1}}{{\bar{F}}_{1}}\times 100$$

All statistical analysis were done using Statistical Analysis System (SAS) (SAS Institute [79]) and graphs generated using Excel (Excel 2013; [80]) Software.

## 4. Conclusions

Overall, highly significant GCA and SCA for all measured traits indicates the importance of additive and non-additive genetic nature in the expression of these traits in both F_1_ and F_2_ generations. Non-additive and additive gene effects were more important in F_1_ and F_2_ generations, except for seed yield under severe water deficit stress, respectively. Therefore, selection programs can be effective in the F_2_ and later generations (F_3_ or F_4_) for improvement of the studied traits under water deficit stress conditions. There was a positive heterosis in coriander for all traits. Even after inbreeding depression, some promising F_2_ crosses displayed good performance and selection in such crosses can provide a better base for future. The progenies of the P_4_ and P_6_ parents displayed better mean performance as compared to their parents and the selection in these crosses provided transgressive gene recombinants for studied traits.

## Figures and Tables

**Table 1:** Combined analysis of variance for phytochemical traits in the F_1_ and F_2_ generations under water treatments.

Source	df	Mean Squares									
		SY		EOC		FOC		EOY		FOY	
		F_1_	F_2_	F_1_	F_2_	F_1_	F_2_	F_1_	F_2_	F_1_	F_2_
Water Treatment (WT)	2	771.31 **	332.34 **	0.53 **	0.193 **	223.12 **	111.27 **	0.008 **	0.004 **	35.08 **	11.53 **
Replication (WT)	6	13.60	12.55	0.43 × 10^−3^	0.33 × 10^−3^	5.02	3.68	0.42 × 10^−3^	0.26 × 10^−3^	0.70	0.53
Genotype (G)	20	45.60 **	21.64 **	0.23 **	0.167 **	102.71 **	63.95 **	0.003 **	0.14 × 10^−2^	2.25 **	0.93 **
G × WT	40	14.75 **	6.27 **	0.02 **	0.015 **	6.13 **	7.10 **	0.6 × 10^−3^ **	0.2 × 10^−3^ **	0.60 **	0.23 **
GCA	5	61.74 **	40.78 **	0.59 **	0.553 **	219.99 **	182.17 **	0.007 **	0.004 **	2.51 **	1.64 **
SCA	15	40.22 **	15.26 **	0.11 **	0.038 **	63.61 **	24.54 **	0.002 **	0.6 × 10^−3^ **	2.16 **	0.69 **
GCA × WT	10	35.18 **	19.27 **	0.02 **	0.022 **	8.65 **	13.54 **	0.001 **	0.6 × 10^−3^ **	1.13 **	0.69 **
SCA × WT	30	7.94 **	1.94 **	0.01 **	0.012 **	5.29 **	4.95 **	0.4 × 10^−3^ **	0.1 × 10^−3^ **	0.42 **	0.08 *
Error	120	1.12	1.10	0.54 × 10^−3^	0.87 × 10^−3^	1.98	2.09	3.87 × 10^−5^	3.1 × 10^−5^	0.05	0.05

** and * are significant at 1% and 5% levels of probability, respectively. Seed yield (SY), essential oil content (EOC), essential oil yield (EOY), fatty oil content (FOC), fatty oil yield (FOY).

**Table 2:** The mean of traits under different irrigation treatments in F_1_ and F_2_ generations of coriander.

Water Treatment	SY		EOC		FOC		EOY		FOY	
	F_1_	F_2_	F_1_	F_2_	F_1_	F_2_	F_1_	F_2_	F_1_	F_2_
Well-Watered	9.19 ^a^	6.74 ^a^	0.351 ^c^	0.337 ^c^	20.59 ^a^	18.35 ^a^	0.035 ^a^	0.023 ^a^	1.88 ^a^	1.22 ^a^
Moderate Water Stressed	4.51 ^b^	3.94 ^b^	0.530 ^a^	0.446 ^a^	18.60 ^b^	17.76 ^b^	0.029 ^b^	0.021 ^a^	0.87 ^b^	0.73 ^b^
Severe Water Stressed	2.35 ^c^	2.18 ^c^	0.477 ^b^	0.377 ^b^	16.83 ^c^	15.81 ^c^	0.013 ^c^	0.009 ^b^	0.43 ^c^	0.37 ^c^

In each column the values with common letters do not differ significantly. Seed yield (SY), essential oil content (EOC), essential oil yield (EOY), fatty oil content (FOC), fatty oil yield (FOY).

**Table 3:** Analysis of variance for combining ability, variance components, and GCA/SCA ratio.

Water Treatment	Estimate	SY		EOC		FOC		EOY		FOY	
		F_1_	F_2_	F_1_	F_2_	F_1_	F_2_	F_1_	F_2_	F_1_	F_2_
Well-Watered	GCA	31.82 **	13.86 **	0.131 **	0.128 **	59.34 **	30.62 **	0.002 **	0.001 **	16.25 **	8.30 **
	SCA	21.19 **	4.85 **	0.018 **	0.014 **	28.44 **	6.88 **	0.001 **	0.26 × 10^−3^ **	26.53 **	6.08 **
	Error	1.65	1.42	0.45 × 10^−3^	0.41 × 10^−3^	2.33	2.19	3.4 × 10^−5^	2.24 × 10^−5^	0.08	0.05
	$\sigma_{g}^{2}$	2.21 ^ns^	0.53 *	0.005 **	0.005 **	1.29 ^ns^	0.99 **	4.5 × 10^−5 ns^	3.64 × 10^−5^ **	0.03 ^ns^	0.004 ^ns^
	$\sigma_{s}^{2}$	18.21 **	1.83 **	0.006 **	0.004 **	8.70 **	1.56 **	0.4 × 10^−3^ **	7.96 × 10^−5^ **	0.64 **	0.08 **
	GCA/SCA	0.12	0.37	0.62	0.68	0.23	0.56	0.18	0.48	0.09	0.10
Moderate Water Stress	GCA	65.85 **	48.31 **	0.323 **	0.307 **	101.93 **	119.15 **	0.006 **	0.003 **	2.873**	2.147**
	SCA	16.30 **	8.64 **	0.074 **	0.041 **	23.03 **	16.00 **	0.001 **	5.3 × 10^−4^ **	0.791 **	0.448 **
	Error	0.90	1.14	0.001	0.001	1.68	1.70	5.3 × 10^−5^	5.0 × 10^−5^	0.049	0.071
	$\sigma_{g}^{2}$	2.06 *	1.65 **	0.010 *	0.011 **	3.29 *	4.30 **	1.8 × 10^−4^ *	1.2 × 10^−4^ *	0.006 *	0.003 **
	$\sigma_{s}^{2}$	5.13 **	2.50 **	0.025 **	0.013 **	7.12 **	4.77 **	4.6 × 10^−4^ **	1.6 × 10^−4^ **	0.009 **	0.003 **
	GCA/SCA	0.45	0.57	0.46	0.62	0.48	0.64	0.44	0.60	0.41	0.53
Severe Water Stress	GCA	13.62 **	11.30 **	0.177 **	0.161 **	76.03 **	59.48 **	6.4 × 10^−4^ **	3.9 × 10^−4^ **	0.68 **	0.48 **
	SCA	4.75 **	3.58 **	0.044 **	0.008 **	22.73 **	11.56 **	2.3 × 10^−4^ **	8.4 × 10^−5^ **	0.20 **	0.12 **
	Error	0.80	0.75	0.001	0.001	1.94	2.37	2.9 10^−5^	2.1 × 10^−5^	0.03	0.03
	$\sigma_{g}^{2}$	0.37 *	0.32 *	0.006 *	0.006 **	2.22 *	2.00 **	1.7 × 10^−5^ *	1.3 × 10^−5^ **	0.02 *	0.02 **
	$\sigma_{s}^{2}$	1.32 **	0.94 **	0.014 **	0.002 **	6.93 **	3.06 **	6.6 × 10^−5^ **	2.1 × 10^−5^ **	0.06 **	0.03 **
	GCA/SCA	0.36	0.40	0.44	0.86	0.39	0.57	0.35	0.55	0.41	0.57

**, * and ^ns^ are significant at 1% and 5% level of probability and not significant, respectively. General combining ability (GCA), specific combining ability (SCA), seed yield (SY), essential oil content (EOC), essential oil yield (EOY), fatty oil content (FOC), fatty oil yield (FOY).

**Table 4:** Coriander genotypes and their characteristics.

Genotype	Parental Code	Characteristics
Commercial	P_1_	Drought susceptible
TN-59-353	P_2_	Relatively drought tolerant
TN-59-80	P_3_	Drought susceptible
TN-59-160	P_4_	Drought tolerant and relatively high yielding
TN-59-158	P_5_	Highly drought susceptible
TN-59-230	P_6_	Highly drought tolerant but low yielding

**Table 5:** Soil properties of different layers of the experimental field.

Soil Depth (cm)	Sand (%)	Silt (%)	Clay (%)	Bulk Density (g cm^−3^)	FC (%)	Organic Matter (%)	pH	EC (dS m^−1^)
0–20	70	15	15	1.2	16.5	1.61	7.75	1.3
20–40	68	18	14	1.4	19	1.45	7.75	1.28
40–60	66	18	16	1.48	15	1.09	7.74	1.26

FC: soil moisture at field capacity; pH: potential of hydrogen; EC: electrical conductivity.

## Data Availability

The data presented in this study are available on request from the corresponding author.
